# Phosphorus adsorption from aqueous solutions using different types of cement: kinetics, isotherms, and mechanisms†

**DOI:** 10.1039/d4ra01929f

**Published:** 2024-05-14

**Authors:** Xingyu Yu, Yongqiang Yang, Hanxiao Zhang, Shijun Wu, Fanrong Chen, Runliang Zhu

**Affiliations:** a CAS Key Laboratory of Mineralogy and Metallogeny, Guangdong Provincial Key Laboratory of Mineral Physics and Materials, Guangzhou Institute of Geochemistry, Chinese Academy of Sciences 511 Kehua Street 510640 Guangzhou China yqyang@gig.ac.cn; b CAS Center for Excellence in Deep Earth Science 511 Kehua Street 510640 Guangzhou China; c University of Chinese Academy of Sciences 19 Yuquan Road 100049 Beijing China

## Abstract

Exploring low-cost and high-performance phosphorus (P) adsorbents is key to controlling P contamination in water. This study evaluated the P adsorption performance of three types of cement: Ordinary Portland cement (OPC), Portland slag cement (PSC), and Portland pozzolana cement (PPC). Furthermore, SEM-EDS, XRD, XPS, and FTIR were employed to reveal the adsorption mechanism. The results showed that the pseudo-second-order model exhibited higher regression coefficients than the pseudo-first-order model, indicating that chemisorption dominated the adsorption process. The Langmuir equation fitted the P adsorption data well, with maximum P adsorption capacities of 245.8, 226.1, and 210.0 mg g^−1^ for OPC, PSC, and PPC at 25 °C, respectively. P adsorption capacities decreased gradually with increasing initial pH and reached their maximum values at pH 3. The anions of F^−^, CO_3_^2−^, and SO_4_^2−^ negatively affected P adsorption due to the competitive adsorption with Ca^2+^. The results of XPS, XRD, and FTIR confirmed that Ca–P precipitates (*i.e.*, hydroxyapatite) were the main removal mechanism. A real domestic sewage experiment showed that 0.6 g L^−1^ OPC effectively reduced the P concentration from 2.4 to below 0.2 mg L^−1^, with a dosage cost of 0.034 $ per ton. This study indicated that cement, as a low-cost and efficient P adsorbent, has great potential for application in removing P from acidic and neutral wastewater.

## Abstract 2

This study reveals the potential of cement as a cost-effective and high-capacity adsorbent for removing phosphorus from acidic and neutral wastewaters.

## Graphical abstract

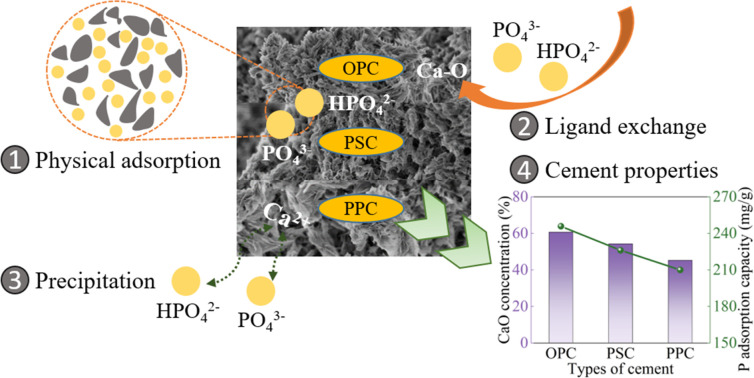

## 1. Introduction

Phosphorus (P) is an essential element vital for the growth and development of living organisms. However, excessive release of P into water bodies, stemming from mining, agricultural, and industrial activities, as well as wastewater discharges, induces eutrophication.^1^ This phenomenon instigates algal blooms that deplete dissolved oxygen, endangering the well-being of aquatic organisms and posing threats to both drinking water supplies and aquatic ecosystems.^1,2^ Numerous studies underscore the significance of P removal in water for effective eutrophication control.^3^ Consequently, an urgent need arises to develop more efficient approaches for the removal of P from wastewater and water bodies.

Traditional P removal methods can be categorized as ecological, biological, physical, and chemical.^4–6^ In contrast to the former three approaches, chemical P removal methods, including precipitation, chemisorption, and ion exchange, stand out due to their operational simplicity, high efficiency and selectivity, cost-effectiveness, and robust stability.^3,4^ Moreover, for deep P removal, chemical adsorption may be the sole low-cost, viable method capable of reducing orthophosphate concentrations to levels less than 0.1 and even below 0.01 mg L^−1^.^7^

The efficacy of chemical adsorption depends on the meticulous selection of adsorbents.^4^ A variety of adsorbents, including natural materials, industrial wastes and by-products, and manufactured materials, have been investigated for P removal. Natural materials, such as zeolite, activated carbon, and quartz sand, exhibit limited P adsorption capacity.^8^ In contrast, industrial wastes and by-products like steel slag, bauxite residue, mine drainage residuals, and red mud demonstrate superior P adsorption capacity owing to the presence of aluminum, iron, and calcium salts.^9–14^ However, the efficacy and potential impacts on ecological systems of employing these adsorbents for P adsorption depend significantly on factors such as organic matter, alkalinity, pH, co-existing ions, and raw materials.^15,16^ A recent research using mine drainage residuals for internal P control has indicated that only negligible amounts of trace metals are introduced into the water column and sediments.^14^ Manufactured materials like lanthanum-containing materials can effectively remove P from wastewater and eutrophic water bodies due to their robust binding with phosphate, leading to the formation of insoluble and redox-stable minerals.^16^ Nevertheless, the potential impacts of these manufactured materials on ecosystems, particularly their potential toxicity to aquatic organisms at low pH levels (<7), warrant careful consideration; furthermore, their manufacturing process is relatively complex and costly.^8,16^ Hence, further research is essential to identify P adsorbents that are cost-effective, efficient, readily available, and safe for large-scale application in practical engineering.

Cement, as the largest man-made product globally, achieved a production volume of 2.4 billion tons in China in 2020.^17^ In addition to its great contribution to the construction industry, cement has been preliminarily identified as having considerable potential in P adsorption.^18,19^ For instance, aluminate cement thermally activated at 600 °C demonstrated a P adsorption capacity of 49.1 mg g^−1^.^19^ The introduction of Portland cement into eutrophic water bodies led to a reduction in total P from 2.2 to 0.2 mg L^−1^.^18^ Limestone, constituting approximately 80 wt%,^17^ is the primary raw material for Portland cement production and likely contributes to its high P adsorption capacity. Currently, the main types of cement include Ordinary Portland cement (OPC), Portland slag cement (PSC), Portland pozzolana cement (PPC), calcium aluminate cement (CAC), and low alkalinity calcium sulfoaluminate cement (CSA), each characterized by distinct compositions. Portland cement primarily consists of lime (63–66% CaO), silica (21–24% SiO_2_), alumina (4–8% Al_2_O_3_), and ferric oxide (1–6% Fe_2_O_3_),^20^ with calcium silicates (C_3_S and C_2_S) as the main reactive phases.^21^ In contrast, the primary active components in CAC and CSA are calcium aluminate and calcium sulfoaluminate, respectively. These compounds endow CAC and CSA with distinctive properties, including low CO_2_ emissions, impermeability, resistance to high temperatures, and high early strength.^21–23^ However, CAC and CSA are produced in smaller volumes and are more expensive than OPC.^23^ Consequently, considering economic and availability factors, Portland cement may be a more cost-effective adsorbent for P removal. Nonetheless, the differences in P adsorption capacities among different Portland cement and their adsorption mechanisms are not fully understood.

In this study, three Portland cements (OPC, PSC, PPC) in China underwent batch experiments to achieve the following objectives: (1) ascertain their P adsorption capacity; (2) evaluate the potential impact of influencing factors (initial pH, adsorbent dosage, and coexisting anions) on P adsorption; (3) identify the primary mechanism governing P adsorption; (4) assess their performance in treating actual domestic sewage. This research deepens our understanding of P-cement interactions and provides practical insights for the application of cement in wastewater treatment and water remediation.

## 2. Materials and methods

### 2.1 Experimental materials

Three powdered cement samples (OPC: grade 42.5R, PSC: grade 32.5R, and PPC: grade 32.5R), with particle sizes less than 74 μm, were obtained from a building materials market in Guangzhou, Guangdong, China. To ensure accuracy and consistency across experiments, the cement samples were oven-dried at 110 °C for 2 hours before each use. All reagents involved in this study, including KH_2_PO_4_, HCl, NaOH, NaSO_4_, NaNO_3_, NaF, NaCl, and NaCO_3_, were of analytical grade. A stock solution containing 1500 mg L^−1^ concentration of phosphate was prepared by dissolving KH_2_PO_4_ in deionized water, which was kept at 4 °C in the dark. The chemical composition and specific surface area of the cement samples were determined using an X-ray fluorescence spectrometer (XRF) and a surface area and porosimetry analyzer (ASAP2460), and the results are detailed in Tables 1 and S1.† The specific surface area of OPC, PSC, and PPC are 1.13, 1.46, and 2.33 m^2^ g^−1^, respectively, which are lower than those of most P adsorbents.

**Table 1:** Chemical composition of OPC, PSC, and PPC (wt%)

Sample	Na_2_O	MgO	Al_2_O_3_	SiO_2_	CaO	TiO_2_	Fe_2_O_3_	LOI	Others
OPC	0.16	4.15	6.48	22.58	60.71	0.19	2.19	2.65	0.89
PSC	0.27	4.75	7.74	24.70	54.28	0.27	1.90	4.94	1.15
PPC	0.23	2.59	10.49	28.00	45.29	0.35	4.53	6.91	1.61

### 2.2 Characterization of cement

The micromorphology of the cement samples was analyzed using a scanning electron microscope (SEM, XLG2, Phenom). Elemental semi-quantitative analysis was conducted with energy dispersive spectroscopy (EDS, XLG2, Phenom). The crystal structure of various cements was examined using X-ray Diffraction (XRD, D8 advance, Bruker) in the range of 10° to 80° (2*θ*). The functional groups of the cement were studied using Fourier transform infrared spectroscopy (FTIR, Spectrum70, Bruker) in the spectral range of 400–4000 cm^−1^. X-ray photoelectron spectroscopy (XPS, K-Alpha) was employed to analyze the valence state, and the obtained binding energy positions were calibrated based on the C peak (284.8 eV).

### 2.3 Batch adsorption experiment

The adsorption experiments were conducted in a batch mode. Unless specified otherwise, the experiments followed these procedures: 0.05 g of adsorbent was weighed and added to centrifuge tubes containing 50 mL of a 100 mg P L^−1^ solution. The reaction proceeded for 6 h on a temperature-controlled shaker at 25 °C and 200 rpm. The supernatant was filtered through a 0.45 μm polyethersulfone membrane (Jinteng), followed by analysis using the ammonium molybdate spectrophotometric method (Chinese National Standard GB 11893-1989). NH_4_^+^-N and COD were quantified using Nessler's reagent spectrophotometric method (Chinese National Standard of HJ 538-2009) and potassium dichromate titration (Chinese National Standard of HJ/T 399-2007), respectively. pH measurements were conducted using a pH meter. Each set of experiments was independently repeated three times in parallel.

Time intervals for the kinetic experiments were set at 0.25, 1, 2, 4, 6, 12, 16, and 24 hours, and the equilibrium adsorption capacity of P was calculated using eqn (1) (eqn (1)). The pseudo-first-order (eqn (2)), pseudo-second-order (eqn (3)), and intra-particle diffusion model (eqn (4)) were employed for fitting.

Equilibrium adsorption capacity:1
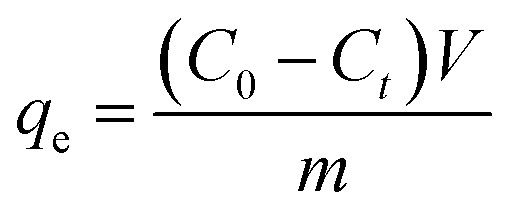


Pseudo-first-order kinetic equation:

2 *q*_*t*_ = *q*_e_(1 − *e*^−*k*_1_*t*^)

Pseudo-second-order kinetic equation:3
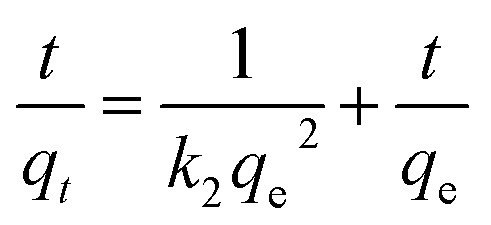


Intra-particle diffusion model:

4 *q*_*t*_ = *k*_*i*_*t*^0.5^ + *C*

where *q*_*t*_ and *q*_e_ represent the adsorption capacity (mg P g^−1^) at time *t* (h) and equilibrium, respectively; *C*_0_ and *C*_*t*_ (mg L^−1^) denote the initial and time *t* concentrations of P, respectively; *V* signifies the volume of solution (L), and *m* is the adsorbent dosage (g); *k*_1_ (1/h) and *k*_2_ (g (mg^−1^ h^−1^)) are the rate constants of kinetic model; *k*_*i*_ (g (mg^−1^ h^−0.5^)) is the rate constant of the intra-particle diffusion model, and *C* is a constant linked to the boundary layer of the adsorbent.

In the isotherm experiment, various initial P concentrations ranging from 5 to 2000 mg L^−1^ were employed, with a shaking duration of 24 h at specific temperature intervals (15, 25, 35 °C). The Langmuir (eqn (5)) and Freundlich (eqn (6)) adsorption isothermal models were applied to fit the adsorption isotherms.5
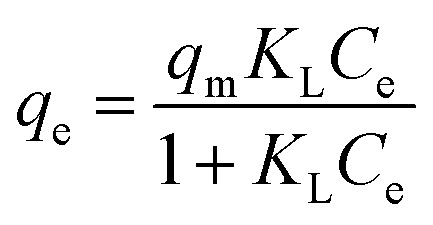

6 *q*_e_ = *K*_F_*C*^1/*n*^_e_

where *q*_e_ (mg P g^−1^) and *C*_e_ (mg L^−1^) represent the adsorption equilibrium capacity and the phosphorus concentration in the equilibrium solution, respectively. *q*_m_ (mg P g^−1^) is the maximum theoretical adsorption capacity estimated by the Langmuir model. *K*_L_ (L mg^−1^) and *K*_F_ (mg g^−1^) represent constants of the Langmuir isothermal model and the Freundlich isothermal model, respectively. *n* is a constant related to temperature, and the higher value indicates the higher adsorption capacity.

The impact of initial pH on P adsorption was investigated across varying pH levels (3, 5, 6, 7, 8, 9, 11) with a constant P concentration of 100 mg L^−1^ and adsorbent dosage of 1 g L^−1^ at 25 °C. To assess the influence of adsorbent dosage on P adsorption, different doses of cement (0.025, 0.05, 0.1, 0.15, 0.2, 0.3, and 0.6 g) were introduced into 50 mL solutions containing 100 mg P L^−1^. The examination of coexisting anions (SO_4_^2−^, NO_3_^−^, F^−^, Cl^−^, and CO_3_^2−^) on P adsorption involved varying background concentrations (0, 10, and 100 mmol L^−1^). Lastly, the adsorption capacity of cement for P from real domestic sewage was conducted to further explore the efficacy of cement in practical application.

### 2.4 Statistical analysis

Statistical significance (*p* < 0.05) of experimental data was performed using one-way ANOVA in SPSS 24.0 (IBM, USA).

## 3. Results and discussion

### 3.1 Adsorption kinetics

**Fig. 1:**
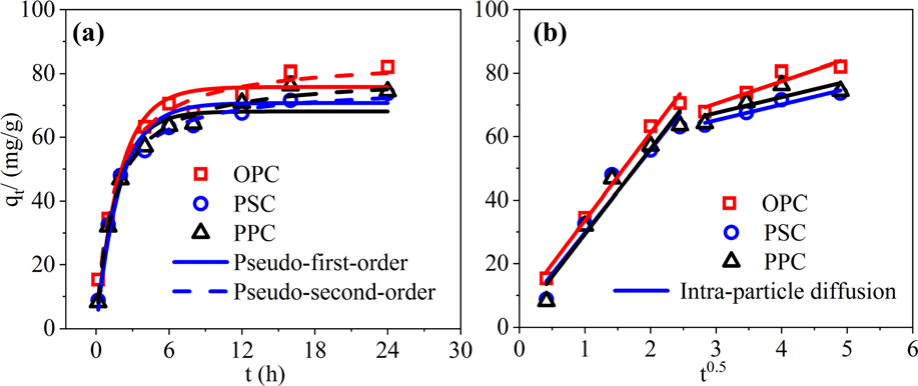
Kinetic models of phosphorus adsorption by cement. (a) Pseudo-first-order kinetic model and pseudo-second-order kinetic model; (b) intra-particle diffusion model. Initial P concentration: 100 mg L^−1^; adsorbent dosage: 1 g L^−1^; temperature: 25 °C; initial pH: 5.

Adsorption kinetics are vital for evaluating temporal variations in adsorption rates and inferring the adsorption mechanism. Fig. 1 demonstrates that a significant proportion of P is adsorbed during the initial phase, with more than 85% occurring within the first 6 h, indicating a higher adsorption rate at the onset. Concurrently, the solution pH rises from an initial value of 5 to around 10 (Fig. S1†). Adsorption equilibrium is attained at approximately 16 h. OPC exhibits a slightly faster adsorption rate than PPC and PSC, as illustrated in Fig. 1. This phenomenon could be linked to the elevated levels of CaO in OPC (Table 1), which may enhance the availability of Ca^2+^ ions and active sites for adsorption during the initial phase. However, the adsorption rate of cement proves significantly slower than that observed for La-based adsorbents,^24,25^ possibly attributed to the gradual release of Ca from cement. To assess the adsorption mechanism concerning P, the pseudo-first-order, pseudo-second-order, and intra-particle diffusion models were applied to fit the kinetic data. Table 2 demonstrates that the pseudo-second-order model exhibits higher regression coefficients for OPC, PSC, and PPC (*R*^2^ > 0.99, *q*_e_ was close to the actual value) than the pseudo-first-order model (*R*^2^ of 0.928, 0.976, 0.943). This suggests that P adsorption on cement is primarily governed by chemisorption. The formation of Ca–P precipitation appears to be the main predominant factor, with physical adsorption playing a lesser role, aligning with findings from previous investigations.^25,26^

**Table 2:** Kinetic adsorption model parameters

Sample 100 mg L^−1^	*q*_exp_/(mg g^−1^)	Pseudo-first-order			Pseudo-second-order			Intra-particle diffusion	
		*k*_1_	*R* ^2^	*q*_e_/(mg g^−1^)	*k*_2_	*R* ^2^	*q*_e_/(mg g^−1^)	*k*_*i*1_	*k*_*i*2_
OPC	82.00	0.00854	0.928	75.78	0.00812	0.997	85.07	27.507	7.023
PSC	73.72	0.00952	0.976	68.08	0.00992	0.999	76.29	26.010	4.966
PPC	74.44	0.00842	0.943	70.73	0.00830	0.998	79.80	26.844	4.976

The rate-controlling step in the overall process was further determined using the intra-particle diffusion model, which includes three mass transfer processes: film diffusion (or external diffusion), intra-particle diffusion (or internal diffusion), and adsorption onto the active sites.^27^Fig. 1b shows that the plot of *q*_*t*_*vs. t*^1/2^ has two linear segments, indicating that the adsorption process has two stages with different adsorption rates (Table 2). The first stage is characterized by a relatively rapid adsorption rate, attributed to the fast diffusion of P onto the cement surface sites, leading to the formation of Ca–P precipitates. The second stage is marked by very low slopes of the intraparticle diffusion segments, which are controlled by intra-particle diffusion, indicating a slow rate of diffusion into the pores of the adsorbent. These results suggest that both film diffusion and intra-particle diffusion occurred during the adsorption of P onto Portland cements.

### 3.2 Adsorption isotherms

**Fig. 2:**
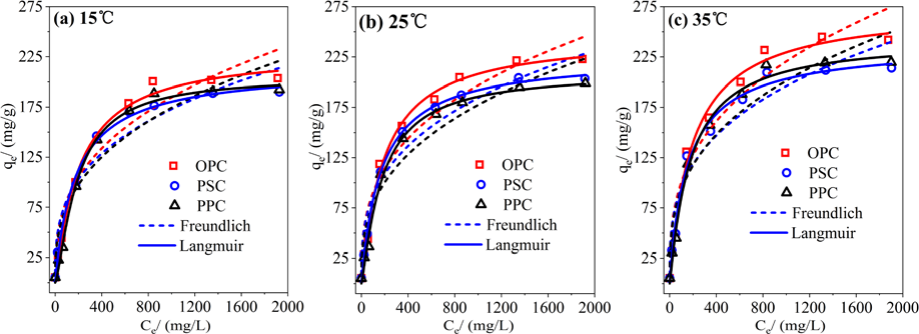
Isotherm models of phosphorus adsorption by cement at different temperatures. (a) Freundlich and Langmuir isotherm models at 15 °C; (b) Freundlich and Langmuir isotherm models at 25 °C; (c) Freundlich and Langmuir isotherm models at 35 °C. Initial pH: 7; adsorbent dosage: 1 g L^−1^; reaction time: 24 h.

The adsorption isotherms of P on cements were investigated to elucidate their adsorption characteristics. The experimental data were fitted using the Langmuir and Freundlich models, as depicted in Fig. 2. The Langmuir model suggests monolayer homogenous adsorption on the adsorbent surface without intermolecular forces, while the Freundlich model represents the multilayer adsorption on heterogamous surfaces.^28,29^ As shown in Table 3, the correlation coefficients of the Langmuir model (*R*^2^ > 0.98) are consistently higher than those of the Freundlich model (0.91 < *R*^2^ < 0.95) at all three temperatures. Consequently, P adsorption on cements can be accurately described by the Langmuir model, which suggests a predominantly monolayer adsorption pattern.

**Table 3:** Isothermal adsorption model parameters at different temperatures

*T* (°C)	Freundlich			Langmuir		
	*K*_F_ (mg g^−1^)	*R* ^2^	1/*n*	*K*_L_ (L mg^−1^)	*R* ^2^	*q*_m_ (mg g^−1^)
OPC-15	16.29	0.939	0.352	0.00322	0.994	230.3
PSC-15	18.57	0.945	0.323	0.00632	0.995	214.5
PPC-15	14.34	0.918	0.362	0.00499	0.996	205.2
OPC-25	18.91	0.936	0.339	0.00432	0.995	245.8
PSC-25	19.22	0.949	0.327	0.00617	0.999	226.1
PPC-25	15.39	0.939	0.354	0.00215	0.994	210.0
OPC-35	20.79	0.944	0.342	0.00561	0.990	274.8
PSC-35	22.34	0.941	0.314	0.00794	0.989	238.5
PPC-35	19.84	0.935	0.335	0.00376	0.992	243.0

Table 3 indicates that the Langmuir model predicts the maximum adsorption capacities for OPC, PSC, and PPC to be 245.8, 226.1, and 210.0 mg g^−1^ at 25 °C, respectively. Notably, these values peak at 35 °C and are lowest at 15 °C, suggesting an endothermic adsorption process that is thermally favorable.^30^ Physisorption typically decreases with rising temperature, while chemisorption tends to increase.^31^ Therefore, the positive correlation between temperature and P adsorption capacity in cements is indicative of chemisorption, aligning with kinetics study results.

A smaller 1/*n* value indicates stronger adsorption affinity, with 1/*n* < 0.5 suggesting easy adsorption, and 1/*n* > 2 indicating difficult dsorption.^32,33^ The consistently low values of 1/*n* for cement imply a strong affinity for P adsorption.

The maximum P adsorption capacities of OPC, PSC, and PPC exhibit a positive correlation with their CaO content, underscoring Ca's pivotal role in P removal. Table 4 compares the P adsorption performance of Ca-based adsorbents with those based on other metals. Notably, Ca-based adsorbents generally exceed the adsorption capacity of other metal-based adsorbents, though they require a longer time to reach equilibrium. This indicates that each adsorbent category possesses distinct advantages. For La-based adsorbents, P adsorption capacity shows a strong correlation with La content but remains independent of specific surface area and pH_pzc_, suggesting precipitation (LaPO_4(s)_) rather than adsorption as the predominant removal mechanism.^16^ Compared to other Ca-based adsorbents, cement demonstrates comparable or superior P adsorption capacities, coupled with the additional benefits of bedding widely available and cost-effective. Hence, cement emerges as a promising candidate for P removal.

**Table 4:** Comparison of phosphorus adsorption capacities of different materials. Experimental conditions: initial P concentration (mg L^−1^); adsorbent dosage (g L^−1^); temperature (°C); adsorption equilibrium capacity (mg g^−1^); pH; equilibrium time (h)

Adsorbent	P Concentration (mg L^−1^)	Adsorbent dosage (g L^−1^)	Temperature (°C)	*q*_e_ (mg g^−1^)	pH	Equilibrium time (h)
MIL-101@SDBS^1^	5–200	1	25	90.8	6	1.5
Mg^2+^ modified pumice^39^	5–20	6	25	17.71	6.5	0.5
Fe–La oxides co-loaded MgO nanosheet^40^	30–70	1	25	38.82	3.15	1.5
La-based adsorbent^25^	10	0.1	25	95.7	2–13	5
La-oxycarbonate based nanotubes^41^	0–750	2.5	25	130.4	4.5	1
Fe_2_O_3_@NH_2_-MIL-101(Fe)^42^	10	0.08	20	36.6	7	0.83
Ca-rich cement mortar^43^	100	1	25	100	7	0.5
CaO-biochar^44^	0–200	0.25	25	231	7	6
Ball-milled Ca-loaded biochar^34^	50–1000	2	25	329	6	3
Sludge Ca-based biochar^45^	120	0.75	25	83.95	7	4
Aluminum cement^19^	2200	2	20	102.9	3–11	12
OPC	5–2000	1	25	245.8	7	24
PSC	5–2000	1	25	226.1	7	24
PPC	5–2000	1	25	210.0	7	24

### 3.3 Effect of initial pH

**Fig. 3:**
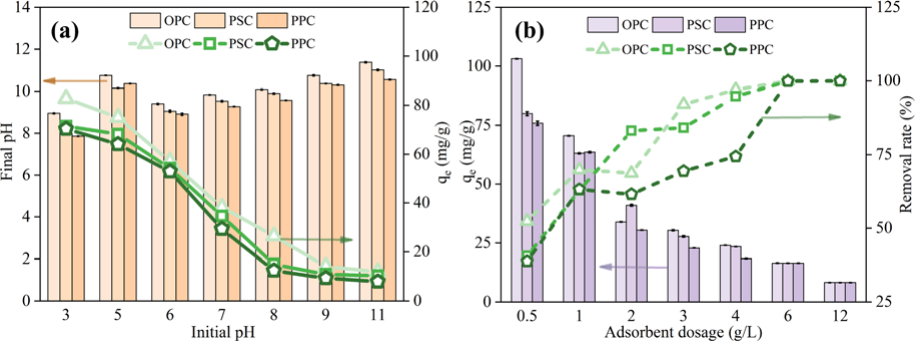
Effect of initial pH (a) on phosphorus adsorption by cement. Initial P concentration: 100 mg L^−1^; adsorbent dosage: 1 g L^−1^; temperature: 25 °C; reaction time: 6 h. Effect of adsorbent dosage (b) on the phosphate adsorption for cement. Initial P concentration: 100 mg L^−1^; temperature: 25 °C; reaction time: 6 h. Error bars represent the standard error of triplicate samples (*n* = 3).

Fig. 3a shows that P adsorption capacities decrease gradually with increasing initial pH, reaching their maximum values of 82.74, 71.46, and 70.37 mg g^−1^ for OPC, PSC, and PPC at pH 3 and their minimum values of 11.88, 10.33, and 7.91 mg g^−1^ at pH 11, respectively. This variation trend is similar to previous studies using Ca-loaded biochar^34^ and cement.^19^ Liu *et al.*^35^ also reported that P removal by calcite was more effective in acidic and strongly basic solutions than in the pH range of 8–11. The final pH exhibits a significant increase compared to the initial pH, particularly at lower initial pH levels, aligning with findings from research using limestone-modified biochar.^26^ This reflects the accelerated dissolution rate of cement in acidic conditions.

The influence of pH on P adsorption is complex, involving surface charge, P speciation, surface site availability, and cement dissolution. The point of zero charge (PZC) for OPC, PSC, and PPC are 12.93, 12.80, and 12.85, respectively (Fig. S2†), consistent with the PZC values reported for hydrated cement pastes^36^ and Ca-PAC.^37^ In this study, both the initial and final pH values were below the pH_pzc_ of cements, indicating a positively charged cement surface, thus facilitating the adsorption of phosphate anions. Predominantly, P exists as H_2_PO_4_^−^ within a pH range of 2.12–7.21 and transitions to HPO_4_^2−^ from 7.21 to 12.36.^38^ As Fig. 3a shows, the final pH mostly falls between 8.0 and 11.0, signifying HPO_4_^2−^ as the main form. This species readily interacts with Ca^2+^, forming various Ca–P compounds such as CaHPO_4_·2H_2_O (brushite, DCPD), Ca_5_(PO_4_)_3_OH (hydroxyapatite HAP), and Ca_3_(PO_4_)_2_ (tricalcium phosphate, TCP) in the presence of hydroxyl groups.^34^ Ho *et al.*^46^ found that Ca(OH)_2_ in concrete fines was the main source of Ca^2+^ at initial pH levels above 4, while calcium silicate and C–S–H also contribute to Ca^2+^ release when the initial pH is 2. At lower initial pH values below 7, an abundance of H^+^ in the solution increases the release of Ca^2+^ from cement, facilitating the formation of Ca–P precipitates. In contrast, at higher initial pH values above 8, P adsorption by cement is impeded by several factors. The alkaline conditions lead to decreased cement dissolution, consequently diminishing Ca^2+^ availability. Moreover, access of OH^−^ competes with HPO_4_^2−^ for adsorption sites on the cement surface, and the high pH induces a negative charge on the cement surface, increasing surface electrostatic repulsion.^47^ Therefore, cement is more effective in treating acidic and neutral wastewaters, whereas its efficiency is reduced in alkaline conditions, corroborating findings from previous studies employing Ca-containing materials.^34,48^

It should be noted that although cement dissolution accelerates at lower pH levels, the release of alkalinity during dissolution quickly neutralizes the pH, thereby slowing the dissolution process. Moreover, the minimal content of heavy metal in the cement ensures that even slight dissolution does not negatively affect the quality of the treated water. However, the potential adverse effects of a higher final pH should be considered in practical applications.

### 3.4 Effect of adsorbent dosage

The impact of cement dosage on P adsorption is depicted in Fig. 3b. Increasing the cement dosage from 0.5 to 6 g L^−1^ results in a significant enhancement in P removal efficiency (*p* < 0.05), rising from approximately 40% to nearly 100%. Consequently, further increasing the dosage to 12 g L^−1^ does not yield any additional improvement in the removal rate. In most dosages, OPC exhibits superior removal efficiencies compared to PSC and PPC, mainly attributable to its higher CaO content. In terms of P adsorption capacity for OPC, a marked decrease in capacity was observed when the dosage was increased from 0.5 to 2 g L^−1^ (*p* < 0.05), dropping from approximately 100 to about 35 mg g^−1^. Subsequently, the capacity further diminished to around 8 mg g^−1^ as the dosage was elevated from 2 to 12 g L^−1^.

Increasing the adsorbent dosage enhances the solid–liquid contact surface area, leading to more adsorption sites and a higher total P adsorption capacity for Portland cement, thus improving P removal efficiency. However, an excessive dosage results in unsaturated or partially saturated adsorption sites, leading to the adsorbent's wastage. Doubling the dosage from 1 to 2 g L^−1^ caused a nearly 50% reduction in the P adsorption capacity of the cements, with only slight improvements in P removal efficiency (Fig. 3b). Consequently, a dosage of 1 g L^−1^ was selected for our experiments considering cost-effectiveness. It is noteworthy that in practical application, the dosage should be tailored to the specific P concentration to achieve more higher P removal rate.

### 3.5 Influence of coexisting anions on P adsorption

**Fig. 4:**
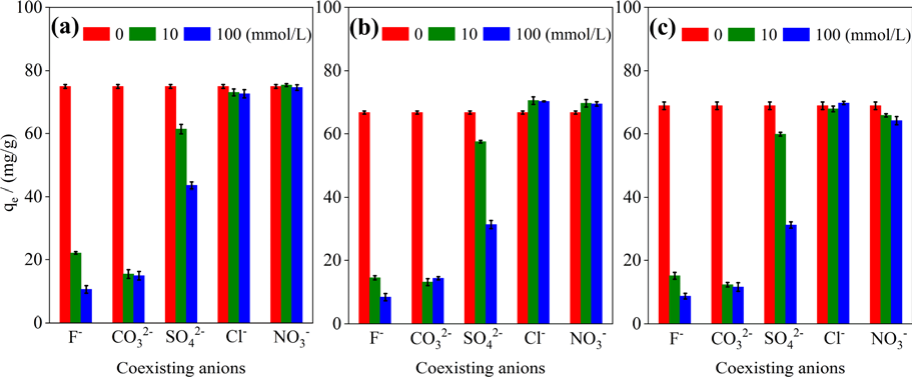
Effect of various anions on phosphorus adsorption by cement (a) OPC; (b) PSC; (c) PPC. Initial P concentration: 100 mg L^−1^; adsorbent dosage: 1 g L^−1^; temperature: 25 °C; reaction time: 6 h. Error bars represent the standard error of triplicate samples (*n* = 3).

To assess the feasibility of using Portland cement for P removal in various water samples, the impact of coexisting anions (F^−^, Cl^−^, NO_3_^−^, CO_3_^2−^, and SO_4_^2−^) on P removal was examined. Fig. 4 shows that both F^−^ and CO_3_^2−^ significantly impede P adsorption, with this inhibition becoming more pronounced as the F^−^ concentration increases from 10 to 100 mmol L^−1^. At a CO_3_^2−^ concentration of 10 mmol L^−1^, the adsorption capacities of OPC, PSC, and PPC decreased to 15.42, 13.08, and 12.25 mg g^−1^, respectively, corresponding to 20.57, 19.60, and 17.78% of their values in the absence of CO_3_^2−^. The solubility constants of CaF_2_ (*K*_sp_ = 3.9 × 10^−11^)^46^ and CaCO_3_ (*K*_sp_ = 2.8 × 10^−9^) are much lower than CaHPO_4_ (*K*_sp_ = 1.0 × 10^−7^).^34^ Therefore, F^−^ and CO_3_^2−^ could compete with HPO_4_^2−^ for Ca^2+^ to form less insoluble precipitation.^49–51^

Despite CaSO_4_ having a higher solubility constant (*K*_sp_ = 9.1 × 10^−6^) than CaHPO_4_, SO_4_^2−^ at a concentration of 100 mmol L^−1^ noticeably inhibits the formation of Ca–P precipitates. This observation aligns with the findings of Lv *et al.*,^52^ which demonstrated a decrease in P removal efficiency to 66.92% in the presence of 100 mg L^−1^ SO_4_^2−^ using Ca-modified granular attapulgite. In contrast, another study found no significant impact on the P adsorption capacity of ball-milled Ca-loaded biochar (BMCa@BC) at the same SO_4_^2−^ concentration.^34^ The distribution of calcium under varying solution pH levels, as calculated using the Hydra-Medusa software, showed that CaSO_4(aq)_ constitutes approximately 40% of the total calcium when the pH is below 9, but this proportion rapidly decreases as the pH increases from 9 to 14.^53^ Therefore, the equilibrium pH emerges as a crucial determinant in regulating the influence of sulfate on the P adsorption by Ca-based adsorbents. As depicted in Fig. S3,† the final pH value for OPC is 9.36, which is higher than those for PSC and PPC (about 8.5), resulting in a diminished impact of SO_4_^2−^ on OPC (Fig. 4). It is noteworthy that sulfate concentrations in domestic sewage typically range from 20 to 60 mg L^−1^,^54^ significantly lower than 10 mmol L^−1^, suggesting that in practical applications of cement for P removal from domestic sewage or surface water, SO_4_^2−^ is unlikely to significantly compromise its P removal efficacy. Cl^−^ and NO_3_^−^ exert negligible influence on P adsorption, primarily because these anions do not form insoluble compounds with Ca^2+^, which is consistent with previous studies.^34^

### 3.6 Adsorption mechanism

#### 3.6.1 SEM-EDS analysis

**Fig. 5:**
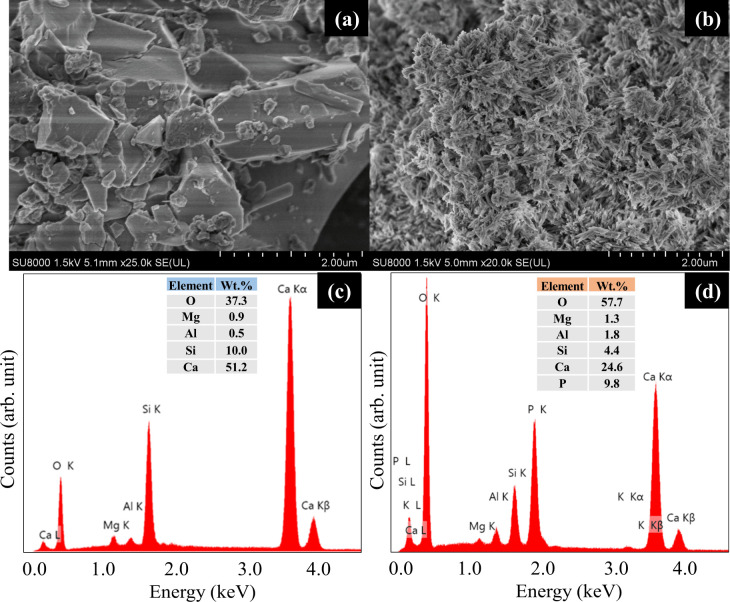
SEM images of OPC (a) before and (b) after P adsorption and EDS spectra of OPC (c) before and (d) after P adsorption.

The SEM-EDS results for OPC are depicted in Fig. 5. Before adsorption, the OPC surface exhibits stacked, irregular block-like structures (Fig. 5a). After adsorption, an abundance of flocculent flower-like clusters emerged (Fig. 5b), likely attributable to cement hydration and subsequent Ca–P precipitation on the surface. This aligns with previous research that correlates such particle aggregation with precipitation process.^31^ Similar features were noted in the SEM images of PSC and PPC pre- and post-adsorption (Fig. S4 and S5†). The EDS spectra of OPC, PSC, and PPC before adsorption exhibit characteristic peaks corresponding to Ca, Al, Si, O, Mg, and Fe (Fig. 5c, S4c, and S5c†), consistent with the XRF results. Notably, a significant increase in P content (9.8%) was discerned on the surface of OPC after adsorption, indicating substantial adsorption of P from the solution onto the cement surface. This observation aligns with the findings of Pan *et al.*,^55^ who reported a notable surge in P content from 0.03% to 3.59% on CaCl_2_-modified buckwheat hulls biochar (BBC1:1-700) following adsorption. Similar trends were documented in previous studies employing CaCl_2_-modified corn stover biochar.^56^ These results collectively validate the efficacy of Ca-containing materials in facilitating P adsorption. However, further analysis is required to determine the specific form of the adsorbed P.

#### 3.6.2 XRD analysis

**Fig. 6:**
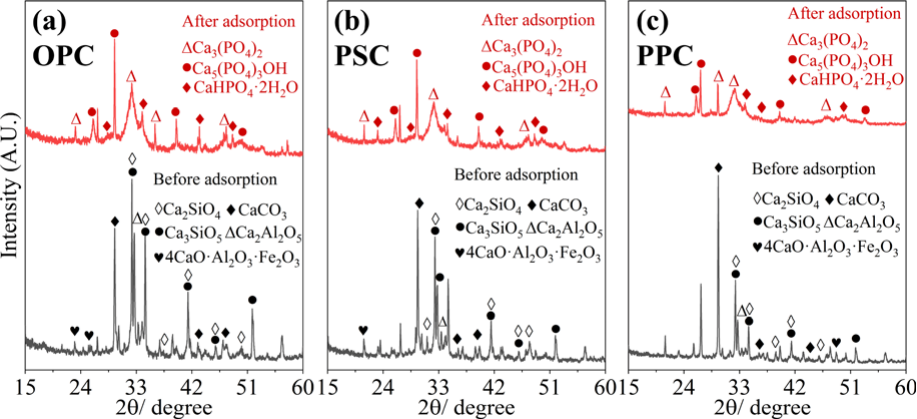
XRD spectra of cement before and after P adsorption. (a) OPC; (b) PSC; (c) PPC.

XRD patterns serve as crucial evidence in elucidating the mechanism of P adsorption on Portland cement. As illustrated in Fig. 6, before adsorption, cements have characteristic peaks of Ca_3_SiO_5_, Ca_2_SiO_4_, CaCO_3_, and Ca_2_Al_2_O_5_, which are common mineral phases found in cement. However, after adsorption, notable changes in the XRD patterns of cements are evident, marked by the notable weakening of peaks corresponding to the common mineral phases (Ca_3_SiO_5_, Ca_2_SiO_4_, *etc.*), and the emergence of new prominent peaks indicative of hydroxyapatite (HAP) (PDF #09-0432), brushite (DCPD) (PDF #09-0077), and tricalcium phosphate (TCP) (PDF #09-0348). The formation of HAP, DCPD, and TCP is attributed to the reaction between PO_4_^3−^/HPO_4_^2−^ and Ca^2+^ ions released from the cement, as described by the eqn (7)–(9). Consequently, chemical precipitation emerges as the main mechanism for P adsorption onto cement, aligning with previous investigations using calcium-biochar.^55,57^

7 3Ca^2+^ + 2PO_4_^3−^ ↔ Ca_3_(PO_4_)_2_↓

8 5Ca^2+^ + 3HPO_4_^2−^ + 4OH^−^ ↔ Ca_5_(PO_4_)_3_OH + 3H_2_O↓

9 Ca^2+^ + HPO_4_^2−^ + 2H_2_O ↔ CaHPO_4_·2H_2_O↓

#### 3.6.3 FTIR and XPS analysis

**Fig. 7:**
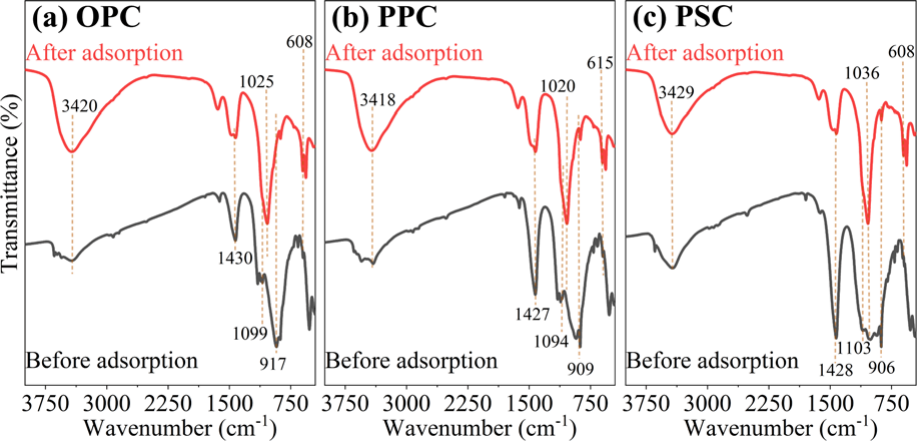
FTIR spectra of cement before and after phosphate adsorption. (a) OPC; (b) PSC; (c) PPC.

The FTIR spectra of cement before and after P adsorption are shown in Fig. 7. After adsorption, the diminished peaks at approximately 1430 cm^−1^, corresponding to the C–O stretching vibrations of CO_3_^2−^, suggest the decomposition of CaCO_3_.^43^ Similarly, the peaks at around 910 cm^−1^, indicative of Ca–O bonds, significantly weakened or disappeared, likely due to hydrolysis of Ca–O and subsequent reaction with P. The Si–O stretching vibrations, represented by peaks near 1100 cm^−1^, maintained low intensity in all samples, consistent with previous research on aluminate cement.^19^ Notably, new sharp peaks around 1025 cm^−1^, indicative of the P–O bond, emerged in the post-adsorption samples. Additionally, all samples exhibited a pronounced O–H bond peak near 3420 cm^−1^, associated with hydroxides^58^ or hydrates. Moreover, the increased intensity of the O–H peak in the adsorbed cements points to the formation of new phases, such as HAP and DCPD. These findings corroborate the successful adsorption of P onto the cement surface through ligand exchange.

**Fig. 8:**
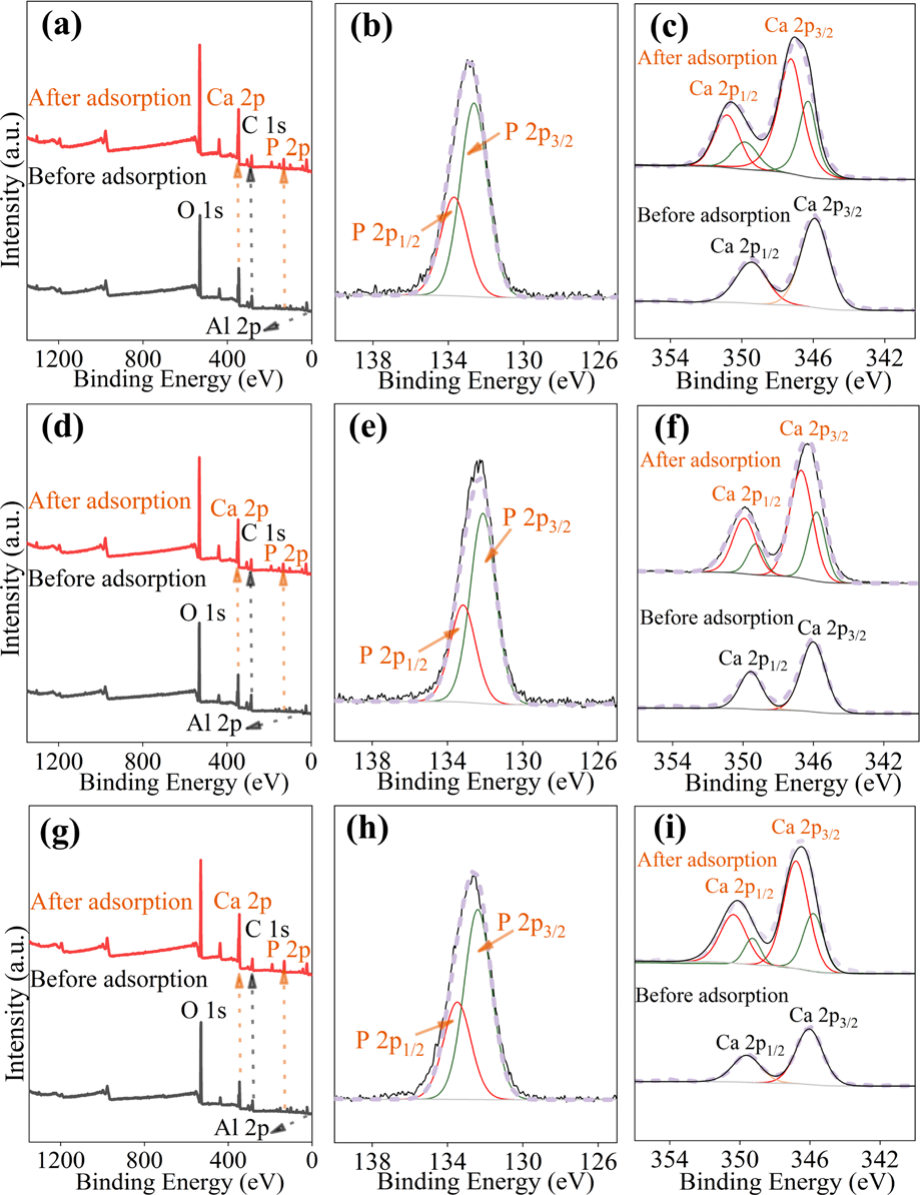
XPS spectra of cement before and after adsorption. XPS full spectra of OPC (a), PSC (d), and PPC (g); P 2p XPS spectra of OPC (b), PSC (e), and PPC (h); Ca 2p XPS spectra of OPC (c), PSC (f), and PPC (i).

The XPS was employed to investigate alterations in the chemical composition and valence states of cements. As depicted in Fig. 8a, d and g, new P 2p peaks emerge around 133 eV after adsorption, indicating the presence of P on the cement surface.^45^ The high-resolution P 2p-XPS spectra delineate two distinct peaks at 132.6 and 133.7 eV, corresponding to PO_4_^3−^ and HPO_4_^2−^, respectively (Fig. 8b, e and h).^59^ Similarly, the high-resolution Ca 2p-XPS spectra exhibit two peaks around 347 eV and 350.5 eV, attributed to Ca 2p_2/3_ and Ca 2p_1/2_, respectively (Fig. 8c, f and i). These results suggest the formation of Ca–P precipitates. However, these chemical precipitates also induce a shift in the binding energy of Ca 2p.^60,61^

The collective findings indicate that the adsorption of P by cement is a multifaceted process involving physical adsorption, chemical precipitation, and ligand exchange. For ligand exchange, phosphate ions displace hydroxyl groups attached to the cement matrix and subsequently bind with Ca^2+^, leading to the formation of Ca–P precipitates, including HAP, DCPD, and TCP, akin to those formed *via* chemical precipitation. Therefore, the primary mechanism underlying P adsorption by cement is identified as the chemical reaction of ligand exchange and surface precipitation.

### 3.7 Application in real domestic sewage

**Fig. 9:**
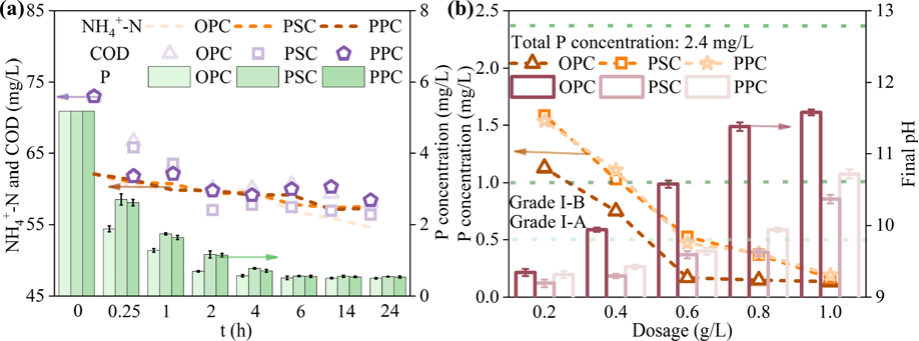
Practical application potential of cement. (a) The removal of P, COD, and NH_4_^+^-N from domestic sewage. Initial concentrations of P, COD, and NH_4_^+^-N were 5.81, 72.98, and 62.13 mg L^−1^, respectively; adsorbent dosage: 1 g L^−1^; temperature: 25 °C. (b) The removal of P from domestic sewage. Initial P concentration: 2.4 mg L^−1^; reaction time: 6 h; temperature: 25 °C.

The adsorption performance of Portland cement in real domestic sewage was investigated to assess its application potential. As depicted in Fig. 9a, the total P concentrations decrease from 5.81 mg L^−1^ to 0.49, 0.55, and 0.54 mg L^−1^ within 6 h with a dosage of 1 g L^−1^, corresponding to removal efficiencies of 91.57, 90.53, and 90.71%, respectively. Notably, the adsorption efficiencies remain stable as the reaction time increases to 24 h, indicating that P adsorption by cement is relatively robust, with no P desorption observed. The P adsorption by Portland cement in synthetic wastewater outperforms that in real domestic sewage (Fig. S6†), possibly due to the more complex matrix of real domestic sewage. Additionally, Portland cement also reduces COD and NH_4_^+^-N concentrations to varying degrees. The introduction of cement creates an aerobic environment in the solution,^18^ which may contribute to COD removal. As depicted in Fig. S7,† the pH gradually increases with reaction time, reaching approximately 11 after 24 hours. This elevated pH facilitates the conversion of NH_4_^+^-N to volatile NH_3_,^62^ thereby aiding in the removal of NH_4_^+^-N.

The cost of P adsorbent is an important factor for evaluating its application prospects. Despite its higher price of 56 $ per ton compared to PSC and PPC (about 49 $ per ton), OPC is chosen for cost estimation due to its superior P removal performance. According to the results above, 1 g of OPC can effectively treat 1 L of domestic sewage with a TP concentration of 5 mg L^−1^, meeting the Chinese Grade I-A discharge standard of pollutants for municipal wastewater treatment plants (GB 18918-2002). Consequently, the average dosage expense for treating 1 ton of domestic sewage using OPC amounts to 0.056 $.

In comparison, the municipal wastewater treatment plants using poly aluminum chloride (PAC) and polyacrylamide (PAM) for P removal reduce TP from around 2 to about 0.2 mg L^−1^, requiring a dosage cost of approximately 0.04 $ per ton (ref. 63–65), which is slightly lower than that of using OPC. However, as shown in Fig. 9b, 0.6 g of OPC can reduce TP from 2.4 to below 0.2 mg L^−1^ in 1 L of domestic sewage, in which case the cost is only about 0.034 $ per ton. Moreover, the price of cement is much lower than those of synthetic P adsorbents used in most studies, such as Ca/Mg-modified biochar,^57^ AT@SiB-X the modification of attapulgite by silicate bacteria.^66^ Considering its high yield, accessibility, and high performance, cement emerges as an economically viable option for P removal from wastewater. However, before large-scale application, several critical issues must be addressed. These include developing methodologies for the extraction and proper disposal of the dosed cement from the treated water. A potential approach involves its utilization as a fertilizer after precipitation, which would necessitate a thorough toxicity evaluation to guarantee plant safety. Additionally, the potential leaching of Ca or other cement constituents into the treated water requires investigation, along with an evaluation of their toxicity. Lastly, the recyclability of cement must be examined, specifically the frequency with which it can be reused, to promote sustainable practices.

## 4. Conclusions

The study demonstrates that OPC, PSC, and PPC are effective adsorbents for P removal, with maximum adsorption capacities of 245.8, 226.1, and 210.0 mg g^−1^ at 25 °C, respectively, according to the Langmuir model. The adsorption kinetics are more accurately described by the pseudo-second-order model, indicating the predominance of chemisorption in the process. Acidic conditions enhance P adsorption by cements, while anions such as F^−^, CO_3_^2−^, and SO_4_^2−^ compete for Ca^2+^, reducing adsorption efficiency. Conversely, Cl^−^ and NO_3_^−^ exhibit negligible impact. The P adsorption mechanism is characterized by the formation of Ca–P precipitates (*i.e.*, HAP, DCPD), supported by XRD, XPS, and FTIR analyses. Experiments with real domestic sewage further confirm the efficacy and cost-efficiency of cement for P removal. While these findings highlight the potential of cement as an adsorbent for P mitigation in acidic and neutral wastewater, the prolonged time to reach adsorption equilibrium and the high pH of the treated water may limit its applicability. For large-scale applications, future research should focus on the recyclability and environmentally sound disposal of used cement. Moreover, investigating the P adsorption potential of eco-friendly cements, such as CAC and CSA, as well as construction waste from various types of cement, is crucial for advancing low-carbon initiatives in water pollution control.

## Author contributions

Xingyu Yu: investigation, methodology, formal analysis, writing – original draft. Yongqiang Yang: conceptualization, formal analysis, project administration, supervision, writing – review & editing. Hanxiao Zhang: investigation. Shijun Wu: writing – review & editing. Fanrong Chen: funding acquisition, supervision. Runliang Zhu: funding acquisition, writing – review & editing.

## Conflicts of interest

There are no conflicts of interest to declare.

## Supplementary Material

RA-014-D4RA01929F-s001
