# Supplementary material for: Phosphorus adsorption from aqueous solutions using different types of cement: kinetics, isotherms, and mechanisms
Source: RSC Adv. 2024 May 14;14(22):15637–46. doi: 10.1039/d4ra01929f (PMC11091795; doi:10.1039/d4ra01929f)
Supplement: RA-014-D4RA01929F-s001 [file RA-014-D4RA01929F-s001.pdf]

Electronic Supplementary Material (ESI) for RSC Advances.
This journal is © The Royal Society of Chemistry 2024

Supplementary Materials for

Phosphorus adsorption from aqueous solutions using different types of cement: Kinetics, isotherms, and mechanisms

Xingyu Yu[a, b, c], *Yongqiang Yang**[a, b], *Hanxiao Zhang*[a, b, c], *Shijun Wu*[a, b], *Fanrong Chen*[a, b], *Runliang Zhu*[a, b]

[a] *CAS Key Laboratory of Mineralogy and Metallogeny & Guangdong Provincial Key Laboratory of Mineral Physics and Materials, Guangzhou Institute of Geochemistry, Chinese Academy of Sciences, 511 Kehua Street, 510640 Guangzhou, China.*

[b] *CAS Center for Excellence in Deep Earth Science, 511 Kehua Street, 510640 Guangzhou, China.*

[c] *University of Chinese Academy of Sciences, 19 Yuquan Road, 100049 Beijing, China.*

**Corresponding author E-mail:yqyang@gig.ac.cn (Yongqiang Yang)*

Supplementary Note 1 | Materials and Methods

Point of zero charge

The point of zero charge (pH_{PZC}) was determined using the pH drift method. The initial pH value of 0.1 M NaCl solution was adjusted to 6, 8, 10, 12, and 13 using 0.1 mol/L NaOH and 0.1 mol/L HCl, with the volume fixed to 20 mL. Subsequently, 0.2 g of sample was added, and the mixture was shaken at 25 °C for 12 hours. The final pH of the supernatant was then recorded. The point at which the difference in pH ($\Delta pH = pH_{Initial} - pH_{Final}$) equals zero represents the zero charge point.

BET surface area

The Micromeritics ASAP2460 Surface Area and Porosimetry Analyzer was used to analyze the N_2 adsorption-desorption isotherms (77.3 K) and BET-specific surface area of the adsorbents. Accurately weighed samples were placed in the test container, following which the adsorbents underwent degassing under vacuum at 200 °C for 2.5 h.

Supplementary Note 2 | Figures and Table

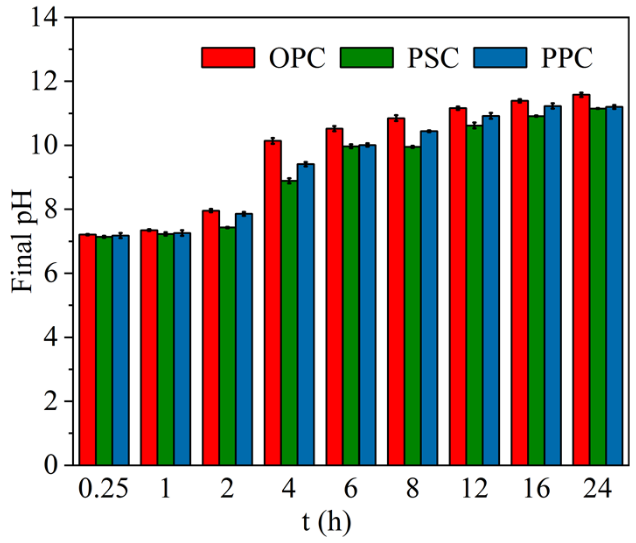

Fig. S1 Temporal variation of solution pH for kinetic models.

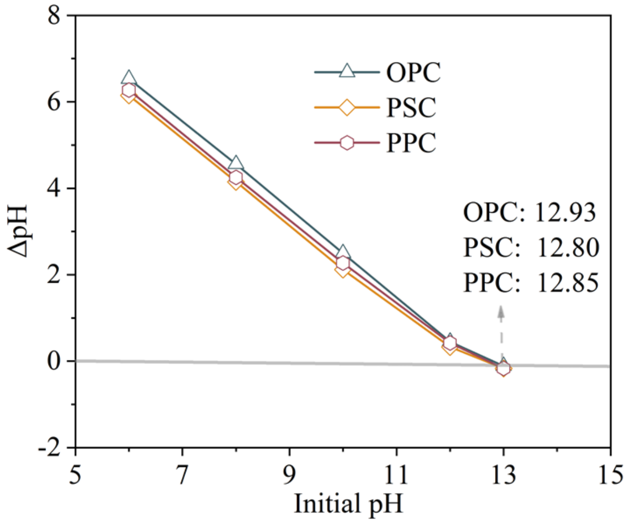

Fig. S2 Plot of ΔpH versus initial pH for pH_{PZC}'s determination of cements. Reaction time: 12 h; Background electrolyte: 0.1 M NaCl; Adsorbent dosage: 10 g/L.

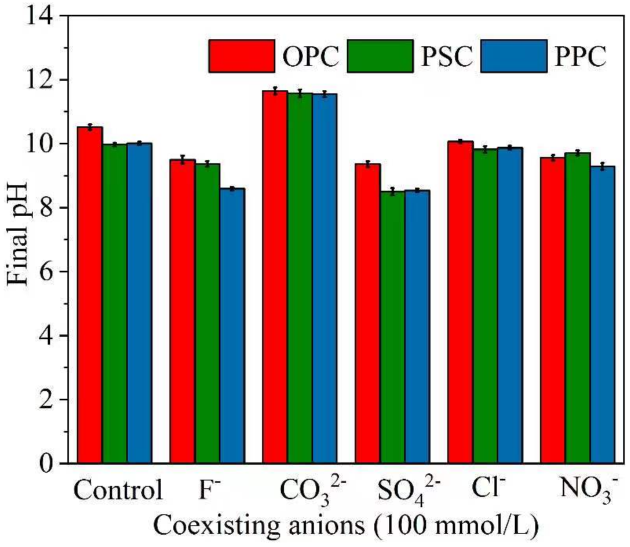

Fig. S3 Variation of solution pH in coexisting anions adsorption experiment.

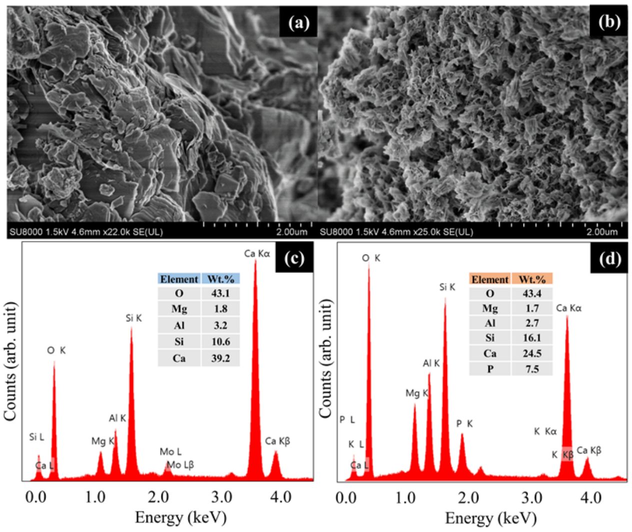

Fig. S4 SEM images of PSC (a) before and (b) after P adsorption and EDS spectra of PSC (c) before and (d) after P adsorption.

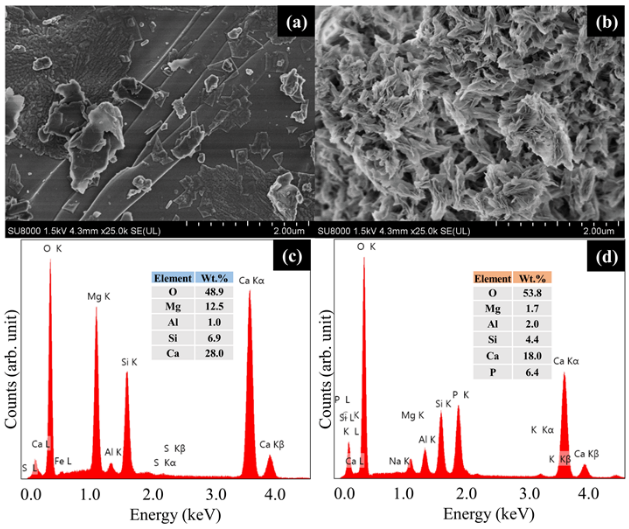

Fig. S5 SEM images of PPC (a) before and (b) after P adsorption and EDS spectra of PPC (c) before and (d) after P adsorption.

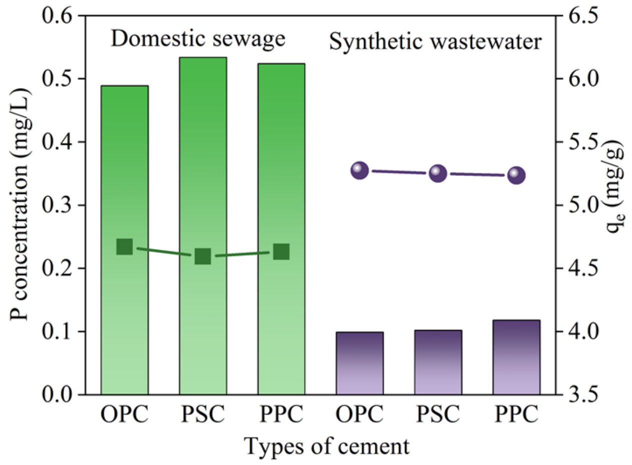

Fig. S6 Effect of water type on P adsorption by cement. Initial P concentration: 5.18 mg/L (domestic sewage) and 5.4 mg/L (synthetic wastewater); Temperature: 25°C; Reaction time: 24 h; Adsorbent dosage: 1 g/L.

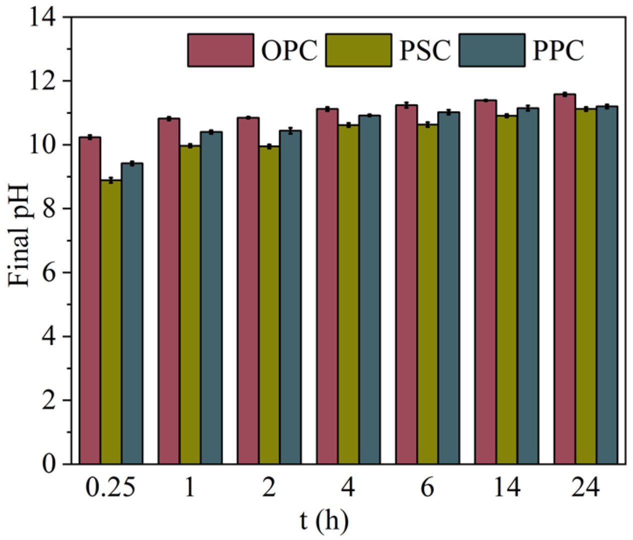

Fig. S7 Temporal variation of solution pH during adsorption experiment with domestic sewage.

Table S1 The specific surface area of cements.

Sample	BET surface area (m^2/g)
OPC	1.13
PSC	1.46
PPC	2.33
